# Evaluating surgical experience in trabeculectomy: Insights from a single-surgeon learning curve

**DOI:** 10.1177/11206721251370056

**Published:** 2025-08-21

**Authors:** Alicja Strzalkowska, Piotr Strzalkowski, Leon Daniello, Esther M Hoffmann, Norbert Pfeiffer, Alexander K Schuster

**Affiliations:** 1 Department of Ophthalmology, 39064 Mainz University Medical Centre of the Johannes Gutenberg, University of Mainz, Mainz, Germany; 2 Department of Ophthalmology, Medical Faculty and University Hospital Düsseldorf – Heinrich Heine University Düsseldorf, Duesseldorf, Germany

**Keywords:** Glaucoma, trabeculectomy, glaucoma surgery, surgical experience, learning curve

## Abstract

**Purpose:**

The aim of our study was to evaluate the outcome of trabeculectomy with respect to surgeon's experience and to explore additional contributing factors.

**Methods:**

This retrospective study was conducted analyzing consecutive single-surgeon cases from October 2019 to June 2022 receiving TE with mitomycin C. Eyes were categorized into five groups based on surgeon experience: E1 for the first 50 TEs, E2 for 51–100, E3 for 101–150, E4 for 151–200, E5 for 201–300. Complete success at one year was defined as an IOP <18 mmHg and 20%-IOP reduction without glaucoma eye drops and revision surgeries.

**Results:**

300 eyes were included (mean age 68.8 ± 13.0 years, 58.3% female). 55.7% had primary open-angle glaucoma. The baseline IOP was 23.4 ± 8.7 mmHg with 3.0 ± 0.9 different antiglaucomatous medication. After 12 months, the complete success for E1 to E5, it was 83.0%, 86.7%, 87.4%, 75.3% and 79.1% (*p* < 0.0001) with IOP 12.8 ± 6.1, 12.8 ± 6.3, 11.5 ± 4.6, 12.9 ± 6.5, 12.9 ± 4.7 mmHg while there was a higher number of NTG cases in E4 and E5 (*p* = 0.04).

**Conclusions:**

Even a novice surgeon can achieve a good surgical outcome within the first 50 trabeculectomy under the guidance of an experienced surgeon. When comparing surgical results over time, it is important to incorporate case entity and severity.

## Introduction

Trabeculectomy is the most common incisional surgical approach for treating glaucoma.^1,2^ It can achieve sufficient reduction in intraocular pressure (IOP) even at low target pressures and without the need for glaucoma medication. The outcome of this surgical technique depends on various factors, such as the type of glaucoma, previous surgeries, concentration of mitomycin C used intraoperatively, or the patient's ethnicity and age. Postoperative care also plays a crucial role, such as laser suturolyses or 5-fluorouracil (5-FU) injections to modify the healing process.

Learning how to perform a well-functioning trabeculectomy is an important part of the surgical curriculum of a glaucoma fellow.^3,4^ The key factor in mastering the technique of trabeculectomy effectively is hands-on mentoring by an experienced glaucoma surgeon, coupled with observation, performance, and postoperative follow-up of an adequate number of cases.^4^ Operating under guidance can allow residents to achieve outcomes in trabeculectomy like those of more experienced surgeons.^5,6^ Developing microsurgical skills requires time and dedication,^7^ and assessing the learning curve is essential to support the safe adoption of new techniques.^8^

The aim of the study was to evaluate the outcome of trabeculectomy with respect to surgeon's experience and to explore additional contributing factors.

## Material and methods

A retrospective study was conducted of all glaucoma patients who underwent trabeculectomy with mitomycin c at the Department of Ophthalmology, University Medical Center of the Johannes Gutenberg-University Mainz between October 2019 and June 2022 by single surgeon (AKS). The patients were categorized into 5 groups based on the surgeon's experience. First 50 trabeculectomy: E1, 51–100: E2, 101–150: E3, 151–200: E4, 201–300: E5. Complete success was defined as IOP <18 mmHg and 20%-IOP reduction without glaucoma eye drops and revision surgeries at 12 months according to TVT-study.^9^ One needling was allowed as a revision surgery in this time frame. Eyes with previous filtrating or other ocular surgeries were also included, no cases of the named time interval were excluded.

### Trabeculectomy – surgical technique

The fornix-based trabeculectomy was performed as previously described.^10^ Initially, the conjunctiva and Tenon's capsule were dissected. One 6 × 6 mm sponge soaked with 0.02 mg MMC in 0.1 ml was placed posteriorly under the Tenon for 3 min. Simultaneously, a shallow groove was created directly behind the former conjunctival insertion to anchor the conjunctiva and Tenon's capsule later on. After 3 min of MMC exposure, intensive rinsing (30 ml) with saline solution was carried out. A 3.5 × 3.5 mm scleral flap of partial thickness was then prepared. A temporal paracentesis was performed, followed by the creation of an anteriorly placed sclero-cornectomy and a peripheral iridectomy. The scleral flap was closed with four 10–0 nylon sutures, consisting of two edges with knots buried under the sclera flap, and two side sutures unburied. The conjunctiva was closed with sutures in a meander-like fashion, as described by Pfeiffer and Grehn.^11^ The presence of a bleb and tightness of the sutures were confirmed by anterior chamber inflation with balanced salt solution.

### Postoperative management

The postoperative eye drop regimen consists of topical antibiotic prophylaxis administered five times a day for 7 days, along with unpreserved prednisolone eye drops six times a day, gradually tapering off over a period of 6 weeks. Additionally, five subconjunctival injections of 5-FU are typically administered, beginning on the second day postoperatively, provided there are no contraindications such as signs of hypotony, corneal erosion, or a positive Seidel test.

Patients are usually assessed during the initial five days in the inpatient clinic. Subsequently, they visit our outpatient clinic one week after discharge, with further follow-up appointments coordinated by their local ophthalmologist.

### Statistics

Statistical analysis was performed using GraphPad Prism10 (Version 10.2.2 (341), GraphPad Software, San Diego, USA) for Mac. For statistical analysis, BCVA was converted in logarithm of the minimum angle of resolution (logMAR) scale. Categorical variables were presented as absolute and relative frequencies, whereas mean and standard deviation were computed for approximately normal-distributed continuous variables, otherwise median and interquartile range. Evaluation of data normality was performed using the D’Agostino and Pearson test. Non-normally distributed continuous data of paired samples were compared by Wilcoxon signed-rank test. For multiple comparisons, non-parametric Kruskal-Wallis test and post hoc Dunn's test were used. All statistical tests were two-sided and *p*-value <0.05 was considered statistically significant.

## Results

### Demographics

The first 300 consecutive trabeculectomies (eyes) from one single surgeon were included in this study. Mean patient age was 68.8 ± 13.0 years and 58.3% were female. Most patients, 55.7%, had primary open-angle glaucoma. The initial MD for the baseline visual field was 9.6 ± 6.7 dB. The mean baseline IOP was 23.4 ± 8.7 mmHg. The number of glaucoma medications was 3.0 ± 0.9. 37% of all eyes had previously undergone a glaucoma operation. The exact demographic for each subgroup based on surgeon's experience (E1-E5) is shown in Table 1.

**Table 1.** Preoperative characteristics of patients divided into 5 groups based on the surgeon's experience E1-E5.

	E1 (*n* = 50)	E2 (*n* = 50)	E3 (*n* = 50)	E4 (*n* = 50)	E5 (*n* = 100)	*P*-Value
Age, years (mean ± SD, range)	70.5 ± 14.1 (23–92)	70.7 ± 10.9 (46–91)	70.4 ± 13.4 (23–88)	66.7 ± 13.3 (20–88)	66.8 ± 12.8 (28–91)	0.04
Female (%)	28 (56)	29 (58)	22 (44)	35 (70)	60 (60)	0.02
Type of Glaucoma (%)						
POAG	32 (64)	28 (56)	25 (50)	27 (54)	53 (53)	0.2
NTG	2 (4)	3 (6)	5 (10)	6 (12)	15 (15)	0.04
PEX	11(22)	17 (34)	15 (30)	10 (20)	18 (18)	0.8
PDG	0 (0)	0 (0)	1 (2)	2 (4)	6 (6)	0.5
Juvenile glaucoma	0 (0)	0 (0)	1 (2)	0 (0)	0 (0)	0.3
Uveitis glaucoma	2 (4)	0 (0)	0 (0)	3 (6)	6 (6)	0.2
Ocular hypertension	0 (0)	0 (0)	1 (2)	0 (0)	0 (0)	0.3
others	3 (6)	2 (4)	1 (2)	1 (2)	1 (1)	0.07
Visual field defect (MD) median (IQR)	9.9 (5.7–17.1)	11.5 (5.5–17.0)	9.8 (4.9–14.7)	7.2 (3.0–14.6)	8.0 (5.0–12.3)	0.02
Mean baseline IOP (mmHg)	26.2 ± 9.5	22.5 ± 8.5	24.2 ± 9.4	22.1 ± 7.1	22.7 ± 8.5	0.02
Number of glaucoma medication at baseline	2.6 ± 0.7	3.4 ± 1.1	3.0 ± 0.5	2.7 ± 1.0	3.3 ± 1.2	0.4
Number of previous glaucoma surgeries (%)	17 (34)	17 (34)	14 (28)	8 (16)	19 (19)	0.1
Number of cataract surgeries (%)	23 (46)	26 (52)	20 (40)	16 (32)	49 (49)	0.06
Number of any other surgeries (ppV, corneal transplantation, etc.) (%)	2 (4)	3 (6)	2 (4)	1 (2)	1 (1)	0.6

E1: first 50 TEs; E2: 51–100 TEs; E3: 101–150 TEs; E4: 151–200 TEs: E5: 201–300 TEs; TE: trabeculectomy.

### Complete success

**Figure 1.**
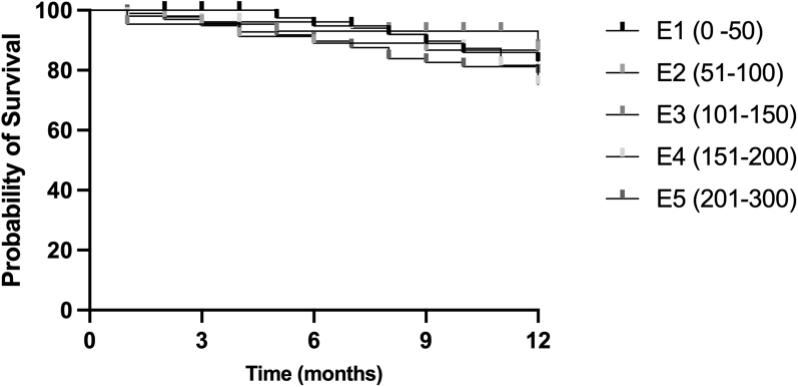
Complete success after trabeculectomy after 12 months of patients divided into 5 groups based on the surgeon's experience E1-E5. E1: first 50 TEs; E2: 51-100 TEs; E3: 101-150 TEs; E4: 151-200 TEs: E5: 201-300 TEs; TE: trabeculectomy.

The complete success rate after 12 months for E1 was: 83.0%, for E2: 86.7% and for E3: 87.4%, for E4: 75.3% and 79.1% for E5, see Figure 1. There was a statistically significant difference between the groups regarding success defined as IOP < 18 mmHg and 20% reduction (*p* < 0.0001, Mantel-Cox test).

### IOP

IOP after 12 months showed no statistically significant difference, regardless of the surgeon's experience and the following results were achieved: E1: 12.8 ± 6.1, E2: 12.8 ± 6.3, E3: 11.5 ± 4.6, E4: 12.9 ± 6.5, E5: 12.9 ± 4.7 mmHg (*p* = 0.47).

### Glaucoma eye drop therapy

A total of 13 patients (4%) from the entire cohort required postoperative glaucoma eye drops. The distribution across the subgroups was as follows: 4% in E1, 2% in E2, 4% in E3, 8% in E4, and 4% in E5. There were no significant differences in the postoperative use of glaucoma eye drops between the groups.

### Analyse of additional contributing factors

#### Type of glaucoma

The most common type of glaucoma was primary open angle glaucoma (POAG) in all groups. NTG patients were rare with 4% of all patients in the E1 and more common in E5 with 15%, *p* = 0.04.

#### Age

The mean age was statistically significant lower in E4 and E5 in comparison to E1-E3. It was accordingly, 66.7 ± 13.3 and 66.8 ± 12.8 vs 70.5 ± 14.1, 70.7 ± 10.9 and 70.4 ± 13.4 years of age, *p* = 0.04.

#### Baseline IOP

**Figure 2.**
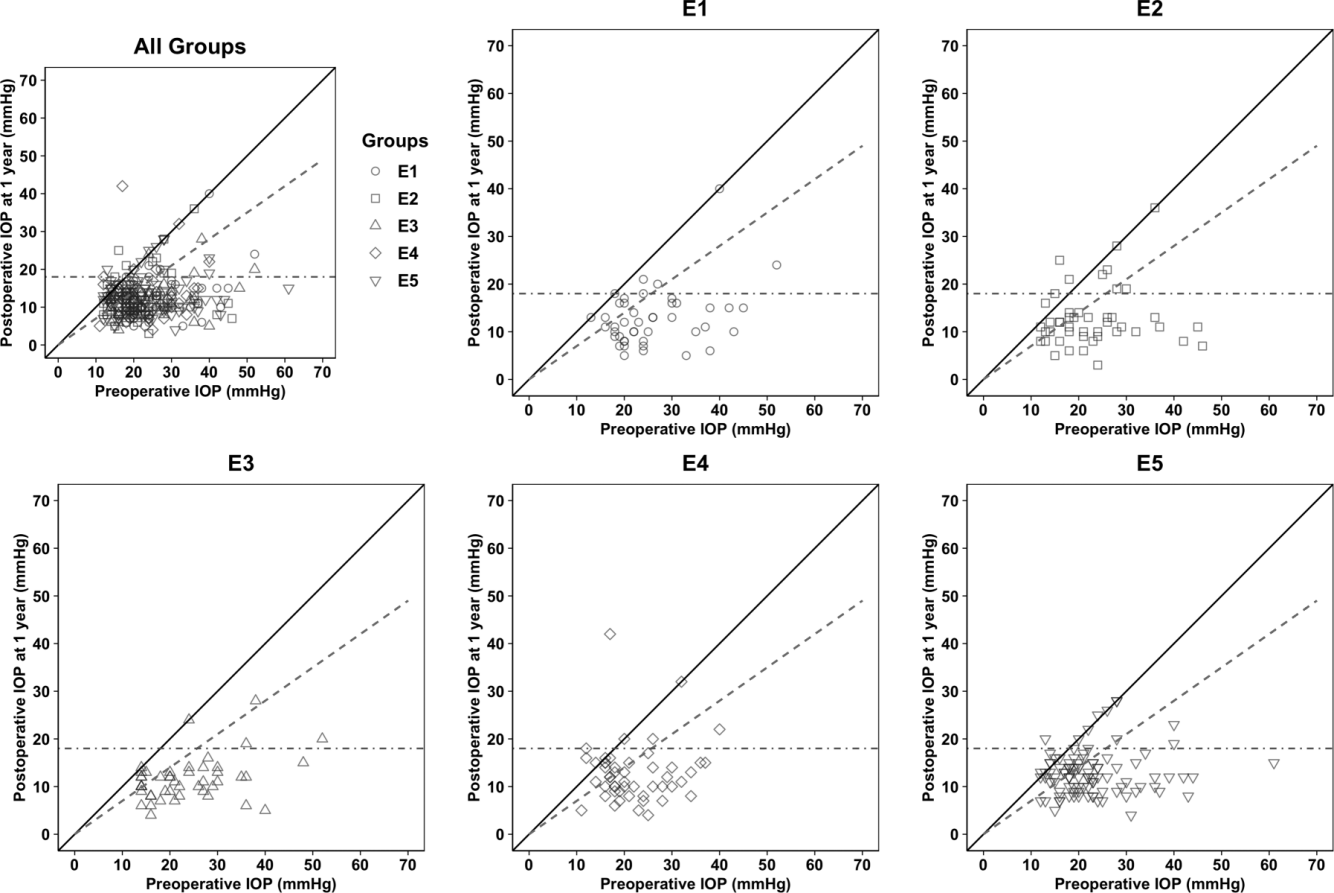
The scatter plot illustrates the relationship between preoperative IOP and postoperative IOP at 12 months of all patients and for each group (E1-E5) separately. IOP: intraocular pressure; E1: first 50 TEs; E2: 51-100 TEs; E3: 101-150 TEs; E4: 151-200 TEs: E5: 201-300; TE: trabeculectomy.

The mean baseline IOP was the highest in E1, 26.2 ± 9.5 mmHg vs 22.5 ± 8.5 mmHg for E2 vs 24.2 ± 9.4 mmHg for E3 vs 22.1 ± 7.1 mmHg for E4 vs 22.7 ± 8.5 mmHg for E5, *p* = 0.02. The scatter plot illustrates the relationship between preoperative IOP and postoperative IOP at 12 months for the entire group and each subgroup E1-E5 separately, see Figure 2.

#### Previous glaucoma operations or laser treatments

A higher proportion of eyes in groups E1–E3 had undergone prior glaucoma surgeries other than trabeculectomy compared to groups E4–E5. However, the difference was not statistically significant: 34% in E1 and E2, 28% in E3, 16% in E4, and 19% in E5 (*p* = 0.1). Across the entire patient cohort, a total of 111 cases with prior ocular surgeries or laser were recorded. Of these, 23 eyes had undergone a trabeculectomy and 18 had received selective laser trabeculoplasty preoperatively. 17 eyes had cyclophotocoagulation, and 10 had undergone cyclocryotherapy. Pars plana vitrectomy was performed in 9 eyes. Furthermore, 34 eyes had received other unspecified preoperative procedures.

#### 5-FUs and suturolyses

The total number of 5-FU subconjunctival injections was 3.72 ± 2.03 for E1, 2.47 ± 1.77 for E2 (*p* = 0.01), 3.77 ± 1.49 for E3, 3.75 ± 1.85 for E4 and 4.01 ± 1.49 for E5.

There was no difference between the groups concerning the number of laser suturolyses was 0.8 ± 1.0 for E1, 0.6 ± 0.8 for E2, 0.6 ± 0.9 for E3, 0.8 ± 1.1 for E4 and 1.0 ± 1.2 for E5.

#### Needling

Within 12 months, 27 patients from the entire study population underwent at least one bleb needling procedure, corresponding to 9% of the total cohort. Additionally, 5 patients (2%) received two bleb needlings.

Bleb needling was performed in 2% of E1, 14% of E2, 6% of E3, 10% of E4, and 12% of E5, with two procedures required in 2% of E5. No statistically significant differences were observed between the groups.

#### Reoperations other than needling

Out of the entire study population, 35 of 300 patients (12%) underwent postoperative revision treatment apart from needling. The majority, 24 patients (8%), underwent an open revision, of them 13 patients (4.3%) because of hypotony and 11 patients (3.7%) due to too high IOP. 8 patients required transconjuntival suture due to hypotony, one patient received a conjunctival suture, another underwent a probe trabeculotomy, and one patient received a new meander suture.

In E1, 7 out of 50 patients (14%) underwent revision surgery. Of these, 2 had transconjunctival suture and 5 underwent open revisions. In E2, 5 patients (10%) received revision procedures: one had a transconjunctival suture, one a conjunctival suture, and 3 underwent open revision. In E3, 6 patients (12%) underwent postoperative revisions. These included one transconjunctival suture, one probe trabeculotomy, and 4 open revisions. In E4, 4 out of 50 patients (8%) required revision. One patient received a transconjunctival suture, and three underwent open revisions. In E5, 13 patients (13%) underwent revision surgery. Among them, 3 had transconjunctival suture, one received a meander suture, and 9 underwent open revisions. There was no statistically significant difference in the type or frequency of revision procedures among the different groups in the study cohort.

## Discussion

In our study, good surgical outcomes —measured by intraocular pressure, glaucoma medication use, and the need for further operation—were achieved by a novice surgeon already within the first 50 trabeculectomies under the guidance of an experienced surgeon.

The complete success rate after 12 months was 83.0% for the initial 50 cases, showed a slight increase in cases 51–100 and 101–150, and then declining in cases 151–200 and 201–300.

Notably, early postoperative IOP (after one week, at discharge) has no influence on the IOP after one year. In our study, the mean postoperative IOP after 12 months was < 13 mmHg for each group and was not statistically significant. Additional data can be found in the supplementary materials.

In the Tube versus Trabeculectomy study by Gedde et al., IOP decreased from 25.6 ± 5.3 to 12.7 ± 5.8 mmHg at one year, with the number of glaucoma medications declined from 3.0 ± 1.2 to 0.5 ± 0.9.^9^ Similarly, in the comparison study with ab externo versus ab interno trabeculectomy conducted by Jea et al., IOP reduced from 26.3 ± 10.9 mmHg to 10.2 ± 4.1 mmHg after two years, with the number of glaucoma medications decreasing from 2.2 ± 1.6 to 0.5 ± 1.0 after ab externo trabeculectomy.^12^ The complete success rate was 76.6% at one year and 66.2% at two years, consistent with our finding.^12^ Stalmans et al. reported a decrease in IOP from 21.2 ± 6 mmHg preoperative to 11.8 ± 4.7 mmHg at 3 months and 12.8 ± 3.0 mmHg at 12 months postoperatively after trabeculectomy.^13^ Additionally, in the study by Strzalkowska et al., IOP decreased from 22.3 ± 5.6 mmHg with 2.7 ± 1.1 glaucoma medications to 11.1 ± 4.4 mmHg with 0.0 ± 0.2 at one month and 12.2 ± 3.4 with 0.1 ± 0.4 at 12 months postoperatively after trabeculectomy.^14^

In studies assessing surgical outcomes, the focus is typically not on the number of procedures performed, but rather on whether an individual is in training or has already attained specialist status. Additionally, data on surgical success are derived from multicenter studies or centers with experienced surgeons. Therefore, it remains unclear how these results can be replicated in real-world settings^3^ and by less experienced surgeons or even by novice. Nevertheless, literature indicates that with appropriate guidance and patient selection, residents can achieve comparable outcomes in trabeculectomy to those of more experienced surgeons.^5,6^

In our study, we observed a decrease in success rates as the surgeon's experience increased, and case selection became less stringent over time, resulting in a higher proportion of normal tension glaucoma (NTG) and younger patients towards the end and patients with higher mean baseline IOP within the first 50 cases (E1).

It is well known that achieving a 20% IOP reduction in NTG cases is particularly challenging, and that younger glaucoma patients tend to have fibrotic trabeculectomy with less filtration function. NTG patients were less common in E1 (4%) and more frequent in E5 (15%) (*p* = 0.04). The mean age in groups E4 and E5 was significantly lower than in E1–E3 (*p* = 0.04).

Baseline IOP was highest in E1, which may have contributed to higher success rates in that group. However, IOP at 12 months did not differ significantly across all 300 cases, consistent with findings from other studies.

Moreover, the success definition in our study was used according to the TVT-study, which is rather a stringent definition in clinical practice and may explain the slight decrease in the success rates over time.

Nevertheless, our findings align with previous studies demonstrating that advanced ophthalmology residents can achieve high surgical success under supervision.^15,16^ For example, Chan et al. reported an 84% success rate for trabeculectomy performed by residents in an academic setting. Similarly, Seider et al. found comparable success rates of 85% for both trabeculectomy and Ex-PRESS shunt procedures performed by residents.^15,16^

Additionally, in the study by Kwong et al. with trabeculectomy, success and complication rates were comparable to those who received trabeculectomies performed by attending physicians.^6^

It should be noted that the success of a trabeculectomy in training settings depends on both the operating surgeons — the trainee and the supervising surgeon. The experience of the supervision surgeon, especially in handling training settings, has a major influence, while on the other side willingness to learn, manual skills including dexterity, ability to follow instructions, stress resilience, and preparation for the procedure play especially a critical role for the trainee. The trainee should be familiar with the surgical steps and, ideally, have practiced them, for instance on pig eyes beforehand. Equally important is the supervisor's willingness to share knowledge, which is a key factor in effective surgical teaching.

The main concern regarding novice surgeons performing trabeculectomies is the potential risk of complications. Overall, the number of patients who required additional IOP-lowering therapy during follow-up was low across the entire cohort and did not differ significantly between the groups. Similarly, the incidence of postoperative complications leading to further surgery was relatively low and showed no statistically significant difference between the groups.

Literature and our own experience suggest that the learning curve for minimal invasive glaucoma surgery (MIGS) is steeper than trabeculectomy, with less challenging intra- and postoperative follow-up routines.^17^

For instance, Al-Mugheiry et al. demonstrated that new procedures, such as Hydrus microstent implantation, can be adopted without compromising outcomes.^8^ Similarly, Marques et al. found that the first six XEN implantations by 10 surgeons yielded IOP and complication outcomes comparable to high-volume centers.^3^ Based on our findings, we recommend reviewing individual cases in the context of surgical success and case selection.

Our study has several limitations. Firstly, it is retrospective in nature. Secondly, it represents the learning curve of a single glaucoma surgeon. Thirdly, data on corneal thickness were not consistently recorded, so its influence on the complete success cannot be analysed. A further limitation is that the second half of all cases were done during the pandemic including more severe and rapid progressive cases as these were primarily sent to our clinic while mild or slowly progressive cases waited for treatment till after the pandemic which may have influenced our results.

It is crucial to encourage novice glaucoma surgeons to continue learning trabeculectomy, ensuring that this important surgical technique is not neglected. Trabeculectomy is the only procedure that can achieve complete success in nearly 60% of cases, even 20 years after the surgery^18^ and is expected to remain the go-to procedure in most advanced and refractory cases in the coming decades.^19^ That is why this procedure should survive.^4^

## Conclusion

This retrospective study aimed to evaluate and compare the success of trabeculectomy at different stages of a single surgeon's training. In this study, the surgeon in training achieved a complete success rate of 83% in the first 50 surgeries under the guidance of an experienced surgeon. The success rate slightly increased to 87% in the next 50 surgeries and remained at 87% in the following 50 surgeries. However, as routine and less strict patient selection were applied in cases 151–200, the success rate dropped to 75%. In the final 100 cases, the success rate stabilized at 79%, which was still lower than the rates in the first three groups. Overall, there was no significant difference in IOP at twelve months for all 300 cases. When comparing surgical results over time, it is important to incorporate case entity and severity.

## Precis

This study evaluated how surgeon's experience affects trabeculectomy outcome. In our study, we observed that even a novice surgeon can achieve a good surgical outcome within the first 50 trabeculectomy under the guidance of an experienced surgeon. Over time, however, case selection became less restrictive, with an increasing number of NTG and younger patients, this may have influenced the success rates.

## Supplemental Material

sj-docx-1-ejo-10.1177_11206721251370056 - Supplemental material for Evaluating surgical experience in trabeculectomy: Insights from a single-surgeon learning curve Supplemental material, sj-docx-1-ejo-10.1177_11206721251370056 for Evaluating surgical experience in trabeculectomy: Insights from a single-surgeon learning curve by Alicja Strzalkowska, Piotr Strzalkowski, Leon Daniello, Esther M Hoffmann, Norbert Pfeiffer and Alexander K Schuster in European Journal of Ophthalmology
