# Supplementary material for: Evaluating surgical experience in trabeculectomy: Insights from a single-surgeon learning curve
Source: Eur J Ophthalmol. 2025 Aug 21;36(1):34–9. doi: 10.1177/11206721251370056 (PMC12722580; doi:10.1177/11206721251370056)
Supplement: sj-docx-1-ejo-10.1177_11206721251370056 - Supplemental material for Evaluating surgical experience in trabeculectomy: Insights from a single-surgeon learning curve [file sj-docx-1-ejo-10.1177_11206721251370056.docx]

Supplement data

Figure 1. The scatter plot illustrates the relationship between postoperative IOP (at discharge) and postoperative IOP at 12 months of all patients; Abbreviations: IOP: intraocular pressure; E1: first 50 TEs; E2: 51-100 TEs; E3: 101-150 TEs; E4: 151-200 TEs: E5: 201-300; TE: trabeculectomy
